# Gas Monitoring in Human Frontal Sinuses–Stability Considerations and Gas Exchange Studies

**DOI:** 10.3390/s21134413

**Published:** 2021-06-28

**Authors:** Han Zhang, Ning Han, Yueyu Lin, Jiawen Huang, Sune Svanberg, Katarina Svanberg

**Affiliations:** 1 National Center for International Research on Green Optoelectronics, South China Normal University, Guangzhou 510006, China; han.zhang@coer-scnu.org (H.Z.); ning.han@coer-scnu.org (N.H.); yueyu.lin@coer-scnu.org (Y.L.); jiawen.huang@coer-scnu.org (J.H.); 2 Guangdong Provincial Key Laboratory of Optical Information Materials and Technology, Center for Optical and Electromagnetic Research, South China Academy of Advanced Optoelectronics, South China Normal University, Guangzhou 510006, China; 3 Lund Laser Centre, Lund University, SE-221 00 Lund, Sweden

**Keywords:** rhinosinusitis, frontal sinuses, GASMAS, optical diagnostics, laser spectroscopy

## Abstract

Acute rhinosinusitis is a common infectious disease, which, in more than 90% of cases, is caused by viruses rather than by bacteria. Even so, antibiotics are often unnecessarily prescribed, and in the long run this contributes to the alarming level of antibiotics resistance. The reason is that there are no good guiding tools for defining the background reason of the infection. One main factor for the clearance of the infection is if there is non-obstructed ventilation from the sinus to the nasal cavity. Gas in Scattering Media Absorption Spectroscopy (GASMAS) has potential for diagnosing this. We have performed a study of frontal sinuses of volunteers with a focus on signal stability and reproducibility over time, accurate oxygen concentration determination, and assessment of gas transport through passages, naturally and after decongestant spray administration. Different from earlier studies on frontal sinuses, water vapor, serving the purpose of oxygen signal normalization, was measured at 818 nm rather than earlier at 937 nm, now closer to the 760 nm oxygen absorption band and thus resulting in more reliable results. In addition, the action of decongestants was objectively demonstrated for the first time. Evaluated oxygen concentration values for left- and right-hand side sinus cavities were found to agree within 0.3%, and a left-right geometrical asymmetry parameter related to anatomical differences was stable within 10%.

## 1. Introduction

The present work relates to spectroscopic studies of the gas contents in human sinuses. We first give a background and motivation for the work. In a following sub-section, the spectroscopic measurement technique is introduced, while in a final section, related previous work is reviewed.

### 1.1. Background

The present work focuses on the further development of a non-invasive laser-spectroscopic technique which could improve the diagnostics and treatment of very common infectious diseases, for which persistent over-prescription of antibiotics has caused an alarming development of bacterial resistance.

Rhinosinusitis is a common infectious disease caused by viruses, bacteria, or more seldom by fungi. The main symptoms are nasal congestion, nasal discharges, headache, and fever, which seriously affect the daily life of patients. Sinusitis occurs all over the world and is a major public health problem. It is the fifth most common infectious diagnosis in open-patient healthcare, affecting about 16% of the United States population each year [1]. Over time, the prevalence of sinusitis has been on the rise, e.g., due to environmental factors such as increased air pollution. According to one U. S. official report, chronic sinusitis is the most common chronic disease among people less than 45 years old, and the second most common chronic disease among people between 45 and 65 years, only out-numbered by hypertension [2]. Children are also affected, even if the disease is easily overlooked and neglected in children. The incidence of sinusitis in children is even higher than that in adults, and may be as high as 32% in young children [3]. Rhinosinusitis imposes a huge clinical and economic burden and affects the quality of life of a large fraction of the population. In 1992, the healthcare sector in the U.S. spent more than 2 billion USD on sinusitis medication [4]. In 1996, the total cost of medical care due to sinusitis was estimated at 5.8 billion USD, out of which 1.8 billion USD (30.6%) was spent on children 12 years of age or younger. This cost estimate does not include lost time for work, and lost productivity [5,6,7].

**Figure 1:**
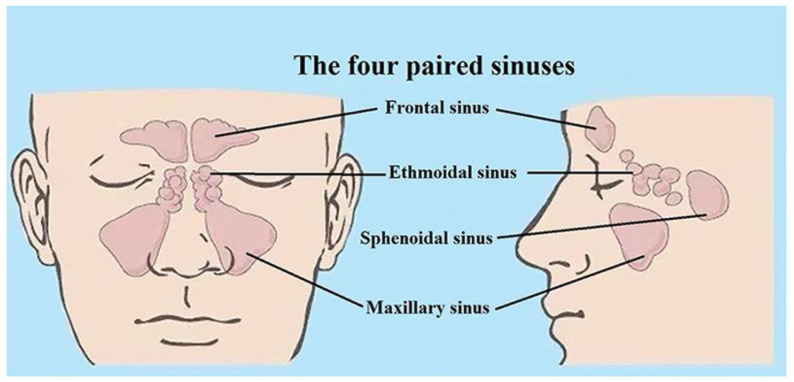
The anatomic locations of the human sinuses. A frontal and a coronal view are shown with the four paired sinuses. Anatomy figure adapted from [9].

The sinuses, which surround the nasal cavity, are gas-filled cavities located in the facial bones and the skull. The mucosa of the nasal cavity continues into the sinuses. This explains why infection of the nasal cavity can cause infection of the sinuses [8]. There are four pairs of paranasal sinuses: the maxillary, the frontal, the ethmoid, and the sphenoid ones, as shown in Figure 1 [9]. They are air-filled, mucosa-lined cavities within the maxillofacial region and skull, and all are communicating with the nasal cavity [10,11]. Sinuses are divided into two groups according to their anatomical location and the opening locations of the sinus connecting passages (the *ostia*). The anterior sinuses are the frontal and the maxillary cavities, all with openings to the middle nasal passage [10,11]. The posterior group of sinuses consists of the sphenoid and the ethmoid sinuses. The former ones open in the sphenoid recess and the latter ones open in the upper nasal passage. The maxillary sinuses are the largest ones [10]. The average volume of the adult maxillary sinus is 10–15 mL. The ethmoid sinus is a honeycomb-like structure within the ethmoid bone, and consists of 3–18 cells. The frontal sinuses are located within the frontal bone. The frontal sinus opening is located at the bottom of the cavity and is funnel-shaped, connecting with the middle nasal passage [12]. Within the sphenoid sinus, there is a septum separating the left and right cavities. All paranasal sinuses are lined with epithelial cells and the connecting passages to the nasal cavity serve for ventilation and transport of mucus. Healthy sinuses are characterized by unimpeded ventilation and normal function of cilia in mucus transport. In the case of viral or bacterial infection, the mucosal tissue becomes swollen, mucus secretion increases, the cilia function becomes impaired, and the nasal passage is partially or completely blocked, which might result in rhinosinusitis [13]. Judged by clinical symptoms, it is difficult to distinguish between viral sinusitis and bacterial sinusitis. Many junior doctors tend to think that sinusitis is an acute bacterial infection, and in 85% to 98% of the cases antibiotics are prescribed [1]. However, in 84% to 91% of the cases the infection is of viral origin [14,15,16]. Even if it is caused by bacteria, the condition disappears in most patients following anti-congestion medication and without antibiotic treatment. The overuse of antibiotics, which has attracted widespread attention, has led to an alarming increase in bacterial antibiotic resistance and medical costs [17,18,19]. It has been estimated that half of the antibiotics use in human medicine is unnecessary, as the infections are caused by viruses [20,21], and the use of antibiotics then only results in resistance development, which is growing rapidly worldwide, especially in Asia, including China, where 25–50% of people carry multidrug-resistant or methicillin-resistant bacteria [22,23,24,25]. A situation, foreseen already in 1945 by the father of antibiotics, Alexander Fleming, is thus quickly developing, possibly constituting a larger threat to humanity than climate change and most pandemics.

The treatment of sinusitis is a huge challenge, where information technology can help in the diagnosis and support decisions on treatment. Accurate anatomical three-dimensional models can be established based on X-ray computed tomography (CT) or magnetic resonance imaging (MRI) data, which can accurately differentiate the inflammatory soft tissue from the obstructing secretions [24]. However, CT and MRI diagnostic procedures are expensive, and often require a special imaging center, which involves travel, delays, and high costs. Thus, simplified diagnostic modalities would be very welcome [26,27]. The recently developed GASMAS (Gas in Scattering Media Absorption Spectroscopy) technique shows considerable promise in assessing gas content of the sinuses. Our study focuses on the further development and quality assurance of this approach in the management of sinusitis.

The present study presents the first GASMAS in vivo recordings of frontal sinuses using a laser emitting light at 760 nm matching one oxygen line combined with recording of water vapor using a laser at 818 nm. The aim of this study was to monitor the stability and reproducibility of the GASMAS signals in the monitoring of frontal sinus cavities. The study also for the first time presents how the opening up of the sinus passages of a rhinitis-affected volunteer, by administration of decongestant medication, can be objectively determined. Our study provides a foundation for a planned future trial in a clinical setting.

### 1.2. Gas in Scattering Media Absorption Spectroscopy

Gas in Scattering Media Absorption Spectroscopy (GASMAS) is a technique used to investigate free gas inside pores or cavities surrounded by a highly light-scattering medium [28]. The GASMAS technique is based on Tunable Diode Laser Absorption Spectroscopy (TDLAS), which uses the interaction between light and gas molecules to determine the concentration of a certain gas. The main concept behind the GASMAS technique is based on the difference between the absorption of light by free molecules in gases and by molecules in liquids and solids. The free gas molecules surrounded by the scattering medium have distinctive sharp absorption signatures, which are approximately 10,000 times narrower than those of the surrounding medium, making it possible to detect very unique signals associated with the gas [29]. The technique emerged in cross-disciplinary interplay between narrow-band spectroscopy of pollution gases in a non-scattering atmosphere, and broad-band spectroscopy of organic constituents in massively scattering human tissue.

When determining the concentration of a gas by using optical spectroscopy, the first step is to calculate the optical path length. In a non-scattering medium, the optical path length is well-defined and measurable. In a scattering medium, the photons pass along an unknown path through gas because of multiple light scattering. Based on the Beer-Lambert law, applied to the case of a scattering medium, the term equivalent average path length can be introduced. It can be determined by measuring the distance the photons must pass through a reference gas (usually the surrounding air), receiving a fractional absorption equivalent to that experienced when light passes through the sample under study [29]. The approach is taken, since only the product of concentration and path length is provided by the Beer-Lambert law. Another way to express the results of a GASMAS measurement is then to give the resulting absorption in terms of percent meter (%*m). As an example, a certain fractional absorption due to, e.g., oxygen of 21 %*m could mean a gas concentration of 21% over a 1 m path, or a gas concentration of 7% over a 3 m path.

As mentioned above, the GASMAS technique is based on the strong difference in the spectral characteristics between free gases and solid substances. In the analysis of gases surrounded by living tissues, the sharp absorption fingerprints of gases (in our case O_2_ and H_2_O) are easily distinguished from the slow changes in absorption of the main tissue constituents in blood, fat, muscle etc. While the physiological gas oxygen is of main interest in human GASMAS studies, water vapor can be used as a convenient reference gas [29,30]. Its concentration, e.g., in the sinuses, depends on the temperature and the relative humidity according to the Arden-Buck equation [31], and a referencing procedure allowing one to obtain the concentration percentage (%) value, rather than the %*m value as provided by GASMAS, will be discussed below. Normalizing the GASMAS oxygen signal to the corresponding one for water vapor is a useful technique, which is further discussed in [32], where also other approaches to path length evaluations in scattering media are discussed.

While our earlier GASMAS studies have been performed with home-built equipment, the instruments used in the present project were manufactured by the company Gasporox AB (Lund, Sweden). The instruments are constructed for monitoring of gas in food packages, and were modified by us for in vivo sinus monitoring. The rationale for introducing these instruments and demonstrating their reliability is to make the transition to clinical studies and acceptance in the hospital setting more realistic. One of the systems was equipped with a laser operating close to 760 nm for oxygen monitoring, while the other one had a laser operating close to 818 nm, and was suitable for water vapor monitoring. The oxygen monitoring system was, for the first time, used to study the gas exchange in the sinus cavities by monitoring the oxygen signal when flushing nitrogen through the nose nostrils in conjunction with administration of anti-congestion medication.

### 1.3. Previous Work on Frontal Sinus Cavities

After a proposal and first demonstration of the possibility to use GASMAS for sinus monitoring [33], a useful laboratory set-up with oxygen as well as water vapor measurement capabilities was integrated. It was used in a study on healthy volunteers, where different geometrical arrangements of light injection and detector positions with regard to the frontal and maxillary sinuses were investigated, and the normalization of the oxygen signal on water vapor was first attempted [34]. While molecular oxygen is always measured using suitable rotational-vibrational transitions in its A-band close to 760 nm, water vapor was, in that exploratory study, probed using prominent lines close to 937 nm. However, at this wavelength liquid water, a main component of tissue, is also strongly absorbing, which is not the case at 760 nm. Because the fate of the water-vapor-probing photons at 937 nm is different from those probing oxygen at 760 nm, from the point of view that the fractions of the total effective path length spent in gas and in the tissue would be different, the normalization procedure can have objections. This is particularly true in a backscattering geometry, where photons because of tissue absorption may less frequently reach the deeper gas-containing layers. The situation is more forgiving in transmission geometry, where the majority of the detected photons for each gas must have passed the gas-containing parts. While the oxygen signal normalization to water vapor thus cannot be expected to work out perfectly for widely separated wavelengths, the results were encouraging, and a clinical study was planned. In preparation for this, a system capable of performing simultaneous monitoring of the two gases was constructed [35], and digital wavelength-modulation spectroscopy was introduced [36]. A subsequent clinical trial involved 40 patients, which were referred to CT imaging of the skull for diagnostics of sinus disorders [37]. It was shown that GASMAS could characterize sinuses (affected by disease, and normal ones) equally well as the standard CT technique, which employs non-desirable ionizing radiation. While this result was very encouraging, information on the stability and reproducibility of the signals over time was still lacking, and no absolute oxygen concentrations were derived. A first study of stability was performed and reported in [38]. However, on this occasion, only equipment capable of water vapor monitoring was available, and thus no oxygen gas assessment or study of sinus ventilation could be performed. The aim of the present study was to overcome earlier limitations and show directions on how to proceed to realistic clinical application. Thus, for the first time, commercial equipment was adapted for clinical use, and the water vapor monitoring was moved to 818 nm, where an about 10-times fainter absorption band is present, which in itself poses a challenge. Very stable signals for the individual gases, and in particular for the signal ratio, which is proportional to the oxygen concentration, could be established. While a sinus ventilation feasibility study by flushing the nostrils with nitrogen was included in [34], only healthy volunteers could be studied. In contrast, in the present work for the first time it could clearly and non-intrusively be demonstrated how decongestant spray could open up initially blocked connecting passages. Recently, an exploratory study, now on human maxillary sinuses in volunteers rather than on frontal sinuses, was performed as an MSc project at Lund University, Sweden [39].

## 2. Study Design

The present study was designed to first establish suitable geometric arrangements for the laser light transmitting fiber and the detector with regard to the frontal sinuses. Measurements on volunteers were performed to study the stability and reproducibility of oxygen and water vapor signals from the frontal sinuses. Ascertaining a reliable evaluation of the oxygen concentration is a further goal facilitated by moving the water-vapor probing wavelength to 818 nm. Studying the influence of administration of decongestant spray in unaffected and rhinitis volunteers was also a key aspect. The stability measurements were performed at different intervals; at the same time every day during seven consecutive days for long-term assessment, and every two hours throughout the day (12 h), starting at 9.30 a.m. and ending at 9.30 p.m., for shorter-time investigation. The sinus ventilation study was performed by observing the influence of nitrogen flushing of the nasal cavity on the measured oxygen signal, in the presence and absence of decongestant spray. During 3 min of flushing through the nostril, the volunteer was asked to breathe normally through the mouth, to reassure that only fresh air with full oxygen content arrived into the lungs.

**Figure 2:**
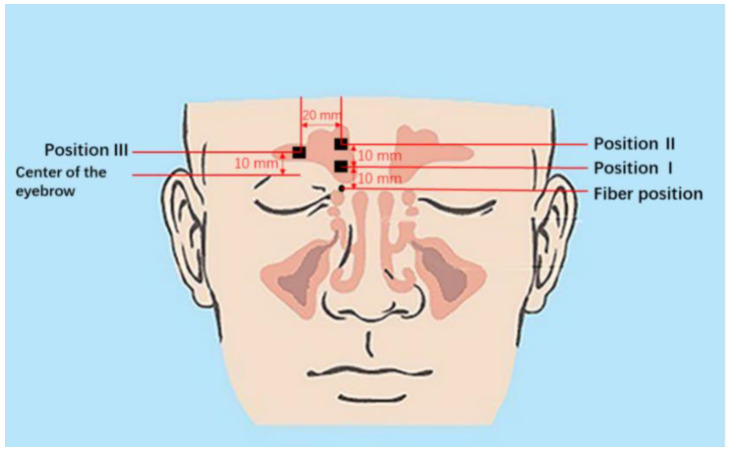
Location sites of the light emitting fiber and the detector. The size of the detector is 10 × 10 mm^2^ and positions relate to the center of the detector. Anatomy figure adapted from [9].

In order to find optimum sinus signals, the detector was put in different positions to observe the strength of the signal (Figure 2). The light emitting fiber was positioned at the base of the nose and emitted the laser vertically upwards as seen in Figure 2. Based on exploratory measurements, it was decided to choose three different measurement geometries: the detector 10 mm above the light source (Position I), 20 mm above the light source (Position II), and 10 mm above the center of the eyebrow (Position III), as shown in Figure 2. A signal sampling time of 2 s was chosen and 30 sets of data at each marked position were recorded.

Normally, even exploratory studies are performed with a certain number of subjects, even if not approaching the numbers needed to ensure significant results, which can be subject to customary Vilcoxon and *p*-value tests. For example, our previous clinical GASMAS study on sinus cavities comprised 40 subjects, and relevant successful tests were performed [37]. In contrast, the present study has a different purpose. Rather than catching an instant situation for numerous subjects at one occasion, with no reference to the time history, the focus was now to see the temporal development in terms of the relative response of the individual gas signals, subject, e.g., to varying mucus fillings in healthy subjects. Further, the prevalence of left-right asymmetries are interesting, as well as the checking the expected oxygen concentration stability, even for changing geometries and mucus filling fractions. This is best done by following individual volunteers over longer time, where repetition in numerous subjects is not expected to bring additional value. Further, we wanted to objectively demonstrate the opening-up of blocked ventilation channels by decongestant administration for the first time, to provide the motivation for a future comparative study of the relative merits of different decongestant formulations.

### 2.1. Volunteers

Four volunteers were included in the study, with the low number motivated above. The volunteers agreed by signing a written consent to take part in the study, which uses a fully non-invasive technique.

Volunteers 1, 2 and 3 had no sign of a common cold or any sinus function aberrations.

Volunteer 4 had allergic rhinitis for a long time, which was accompanied by symptoms such as blocked nasal passages.

### 2.2. Gas Monitoring Systems

**Figure 3:**
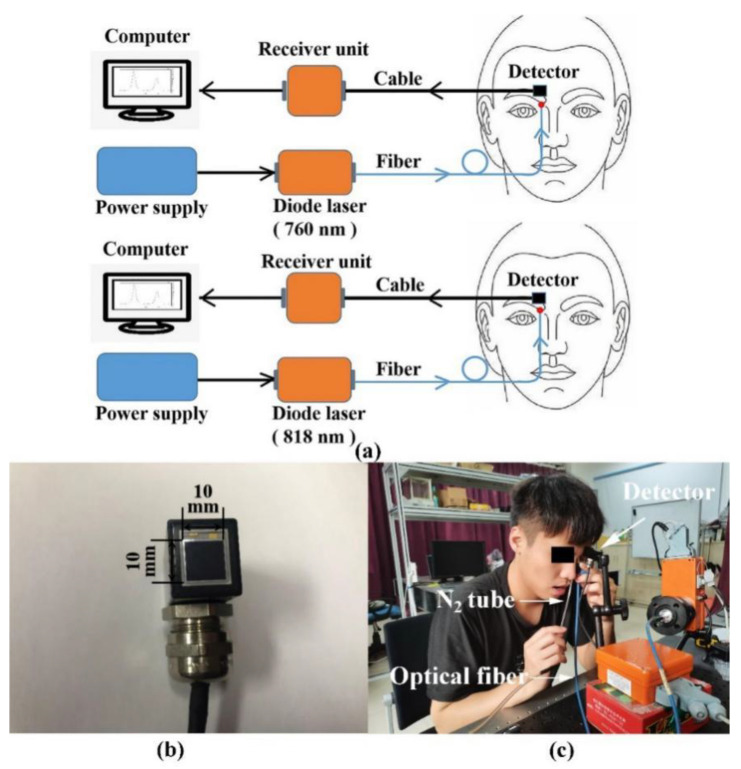
(**a**) Diagram of the arrangement of the two GASMAS systems with lasers, emitting light at 760 nm and 818 nm, respectively. (**b**) Photograph of the detector. (**c**) Photograph of the systems during measurement.

**Figure 4:**
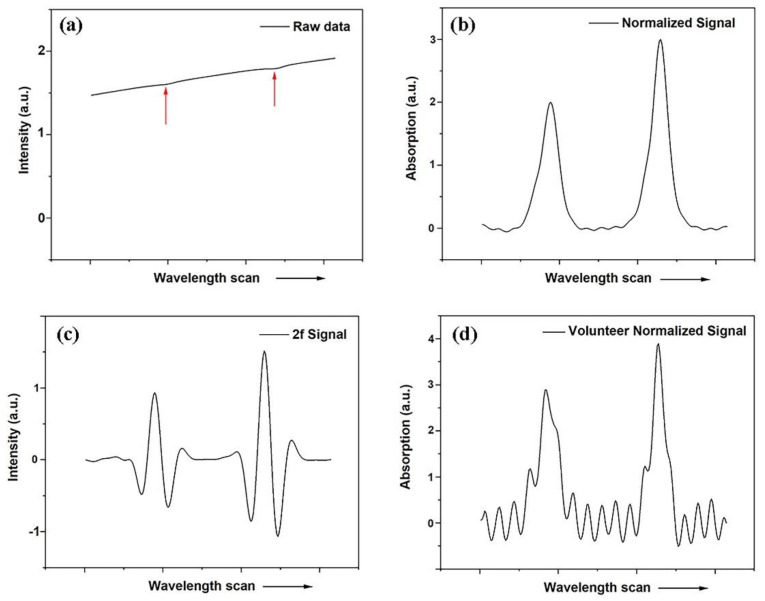
(**a**) Detector output when the laser frequency is ramped through two oxygen line positions. The sample is a 2 cm thick piece of polystyrene foam. (**b**) Isolated oxygen absorption signals derived from the data in (**a**). (**c**) The second derivative of the data shown in (**b**). (**d**) Typical recording of oxygen signals recorded through a human frontal sinus. The fitted fractional gas absorption is in our instrument expressed in the unit percent meter (%*m), since only the product of concentration and interrogated gas distance is determined.

In contrast to earlier work, which employed laboratory-designed systems working with wavelength-modulation spectroscopy techniques [36], two modified commercial GASMAS systems supplied by Gasporox AB (Lund, Sweden) were used in the present study. These systems were not designed for medical use, but rather for non-intrusive monitoring of gas in food packages. Accordingly, the interface to fit the present purpose of frontal sinus exploratory studies was modified; see Figure 2 and Figure 3. The systems were used for molecular oxygen and water vapor monitoring. The semiconductor lasers employed in the systems had output powers of about 5 mW. The systems utilize direct detection of the gas absorptive imprints. Figure 3a shows the experimental arrangement, and Figure 3b is a photograph of the detector. Finally, Figure 3c is a photograph of the systems applied in measurement on a volunteer. An optical fiber transmitting probe carrying the laser light to the frontal sinuses was placed distally at the edge of the orbit, pointing up towards the frontal bone with the sinus cavity. The detecting photodiode was placed on to the frontal bone above the light injection point at positions indicated in Figure 2. The arrangement can be considered to correspond to a modified back-scattering geometry, with vulnerability to differential absorption effects in the tissue matrix, as discussed above. Figure 4a shows a raw recording of the detected light when the transmitting fiber and the detector were placed on opposite sides of a 2 cm thick test sample of strongly scattering polystyrene foam. The intensity rise of the ramp is due to the wavelength tuning, accomplished by increasing the driving current. Two faint absorption lines are indicated by arrows. As can be seen, signals are very weak even for a sample with a long diffuse path length through oxygen. This reflects the fact that these oxygen transitions are forbidden in the electric-dipole approximation in the theory of radiation, and occur weakly only because of higher-order effects.

Figure 4b shows the two absorption lines isolated after subtracting a fitted background. It is well-known that 1/f noise can effectively be suppressed by operating at a high frequency f. In wavelength modulation spectroscopy [36], employed in our earlier studies [33,34,35,37,38], the wavelength was modulated appropriately to generate a derivative-shaped signal due to the absorptive imprint. Frequently, the second derivative is used in the signal evaluation. In contrast, direct absorption monitoring was employed in the present work, still with good performance. Figure 4c displays the second derivative of the data shown in Figure 4b, to provide a connection to the earlier work. Finally, Figure 4d shows a typical recording of oxygen from a sinus cavity.

The retrieved absorption data generated with the system is the concentration *C* of a gas in the cavity times the effective scattering path length *L* in gas through the sinuses. As explained above, the data recorded by a GASMAS set-up can after proper calibration be expressed as:

(1) $$\begin{array}{c} Signal\left(\%*m\right)=C\left(\%\right)*L\left(m\right) \end{array}$$

When monitoring the signal with the 760 nm system, the oxygen signal ($Signal_{\left(O_{2}\right)}$) is obtained, and similarly when using the 818 nm system, the water vapor signal ($Signal_{\left(H_{2}O\right)}$) is recorded. As the water vapor concentration $C_{\left(H_{2}O\right)}$ is known, provided the temperature and relative humidity are known [31], the water vapor concentration can be used for normalization, and the oxygen concentration $C_{\left(O_{2}\right)}\;$ can be derived by dividing Equation (1) for oxygen and water vapor:

(2) $$\begin{array}{c} \frac{Signal_{\left(O_{2}\right)}}{Signal_{\left(H_{2}O\right)}}=\frac{C_{\left(O_{2}\right)}*L_{\left(O_{2}\right)}}{C_{\left(H_{2}O\right)}*L_{\left(H_{2}O\right)}} \end{array}$$

If the wavelengths used for the two gases would be very close to each other, the scattering and absorption through the sample would be the same, and the path length *L* through gas would be the same $\left(L_{\left(O_{2}\right)}=L_{\left(H_{2}O\right)}\right)$. Then, the path length would be eliminated from the equation:

(3) $$\begin{array}{c} C_{\left(O_{2}\right)}=\frac{Signal_{\left(O_{2}\right)}}{Signal_{\left(H_{2}O\right)}}C_{\left(H_{2}O\right)} \end{array}$$

Since 818 nm is now quite close to 760 nm, an assumption of equal path length is quite reasonable. Then, the two measured signals and the water vapor concentration (known from the Arden-Buck relation and the known temperature) can be used to evaluate the oxygen concentration according to Equation (3).

## 3. Measurements and Results

We divide this section into four subsections. First, we describe the instrument calibration, followed by the presentation of signal stability and reproducibility. Then, in Section 3.3, the oxygen-concentration retrieval results are presented. In a final subsection, the results regarding gas transport through the ostia are given.

### 3.1. Instrument Calibration

Our instruments display the measured gas signal in units of %*m, reflecting the fact that only the product of concentration and path length is determined in measurements based on the Beer-Lambert law. The oxygen instrument is factory calibrated against the 21% ambient oxygen concentration for its standard use in food-package monitoring, and the %*m reading based on the signal fit to the recorded oxygen absorption lines apply directly to the GASMAS case. However, the situation is different for water vapor, which is not a normal application for this type of instruments. Air temperature, as well as relative humidity, affects the calibration, which was performed by sensing a 1 m air distance of 25 °C room temperature and a measured relative humidity of 50%. Using the Arden-Buck relation [31], it was found that the relative %*m value given as the signal output of the water vapor instrument should be multiplied by a factor of 1.48 to yield absolute %*m values. The primary data for water vapor recorded in our subsequent studies and displayed in figures are the relative values, subject to the calibration factor when true oxygen concentrations are evaluated in the normalization to water vapor data, as described by Equations (2) and (3).

### 3.2. Signal Stability and Reproducibility

Two healthy volunteers were involved in our study of GASMAS signal stability and reproducibility.

#### 3.2.1. Monitoring during One Week

The one-week signal stability monitoring study was performed with two clinically judged healthy volunteers without any sign of upper respiratory infection. The measurements were scheduled at the same time during seven consecutive days. Every measurement started with monitoring of the left side, followed by recordings of the same corresponding locations of the right side. The whole measurement procedure on both sides lasted for approximately 15 min. Arrangements were made to ensure full eye safety for volunteers and investigators.

Volunteer 1

**Figure 5:**
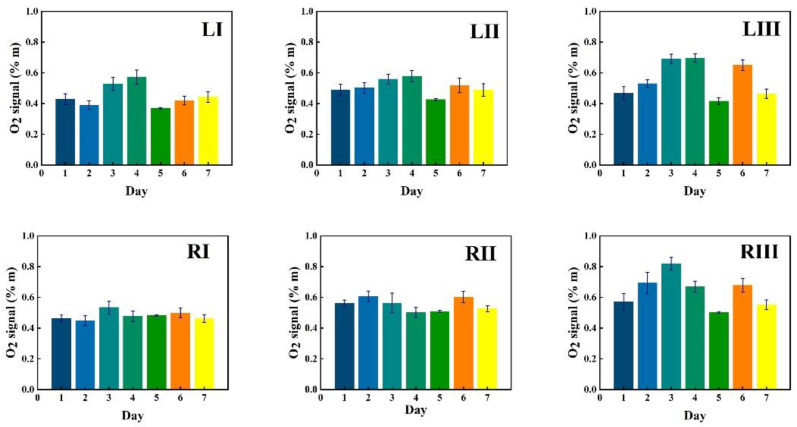
The changes of measured oxygen signal at different locations (according to Figure 2) for volunteer 1 during one week, together with the standard deviation.

Data in Figure 5 show measurement results for a first volunteer as recorded at the six chosen locations during one week (seven consecutive days), marked with bars of different color. The data in the figures are given in six sequences, each for the same time span starting with the I, II and III locations for the left frontal sinus, followed by the I, II and III locations for the right frontal sinus. The recorded %*m values from the instruments are plotted with one standard deviation of the 30 consecutive recordings, each lasting 2 s.

**Figure 6:**
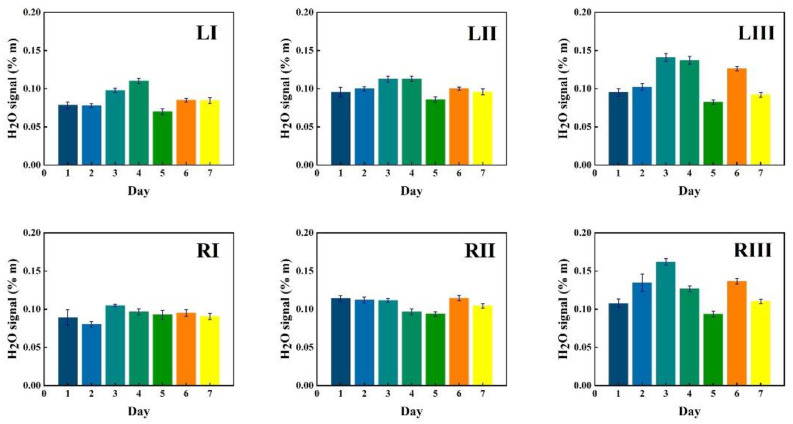
The changes of measured water vapor signal at different locations for volunteer 1 during one week, together with the standard deviation.

**Figure 7:**
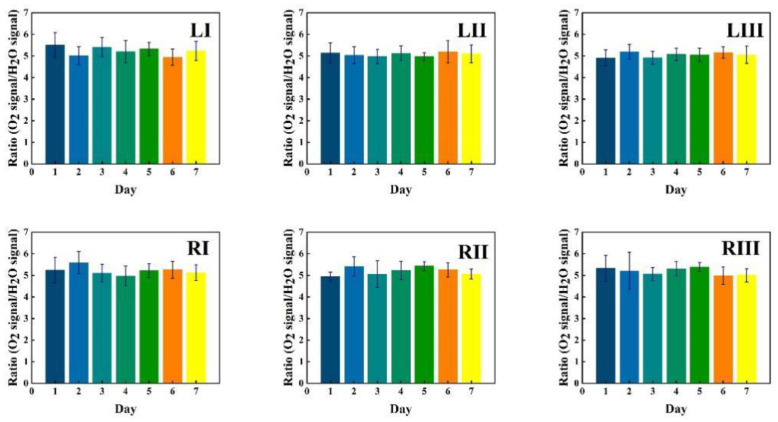
The ratio of measured oxygen and water vapor signals at different locations for volunteer 1 during one week, together with the standard deviation.

Corresponding data for water vapor are shown in Figure 6, while the ratio of the oxygen and water vapor results (from Figure 5 and Figure 6, respectively) is plotted in Figure 7. Day-to-day variations in both gas signals are moderate, and believed to reflect slightly varying mucus fillings. Recorded differences between different locations are persistent over time for both gases. With minor deviations, it is observed that for both gases, the signals increase until day 3–4, then exhibit a minimum for day 5, again to rise day 6 and fall back day 7. The average signal value going from location I to II, and I to III increases persistently and reflects the longer pathway in gas when injection-detection distance is increased. The oxygen/water vapor ratio data, which according to Equation (2) are proportional to the oxygen concentration, are closely similar for all locations and over time, reflecting that the behavior is the same for oxygen and water vapor.

Volunteer 2

**Figure 8:**
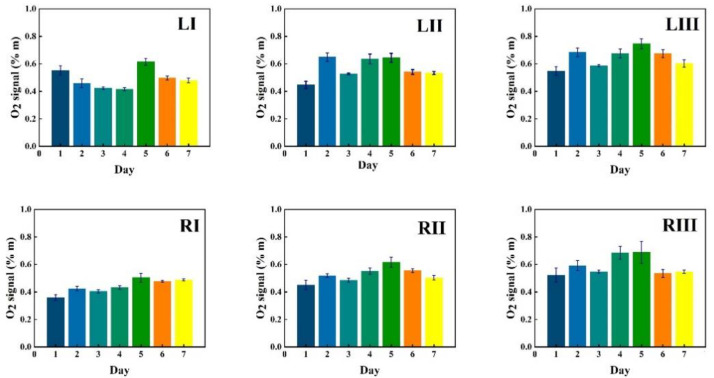
The changes of measured oxygen signal at different locations for volunteer 2 during one week, together with the standard deviation.

**Figure 9:**
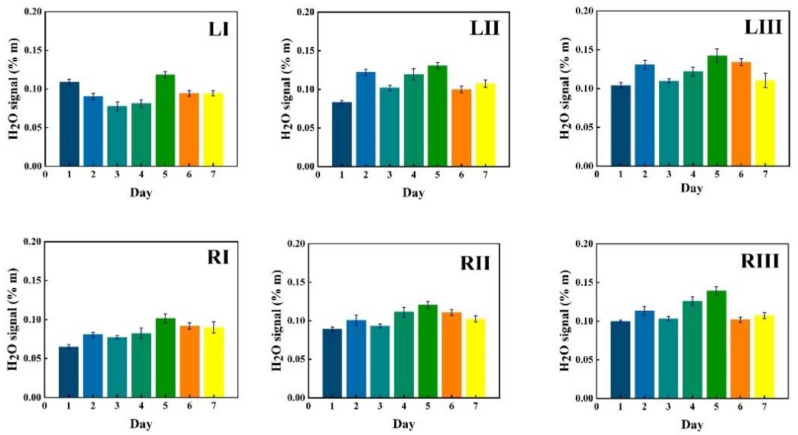
The changes of measured water vapor signal at different locations for volunteer 2 during one week, together with the standard deviation.

**Figure 10:**
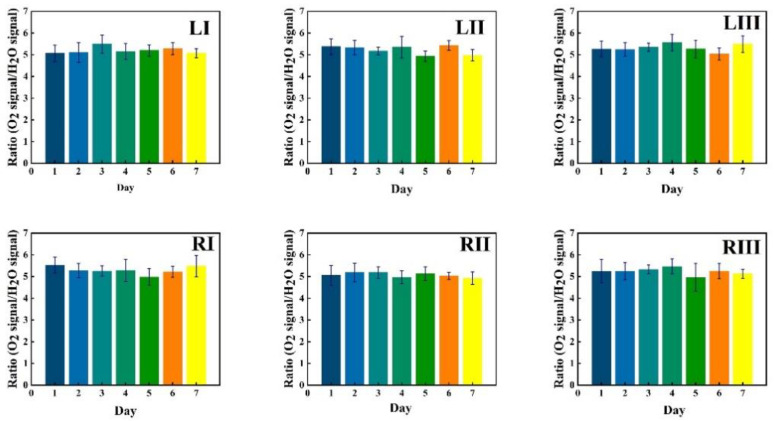
The ratio of measured oxygen and water vapor signals at different locations for volunteer 2 during one week, together with the standard deviation.

The same type of data as shown in Figure 5, Figure 6 and Figure 7 for volunteer 1 were recorded for volunteer 2, and the results are shown in Figure 8, Figure 9 and Figure 10.

Just as for volunteer 1, the data from volunteer 2 show similar consistency in value trends between locations and as a function of time. With minor deviations it is found that for both gases and for the different measurement locations, the signals increase to reach a maximum at day 5, then fall again. The average signal value going from location I to II, and I to III again increases persistently, reflecting the detector positioning further away from the light injection point. Reflecting the temporal co-variation of the signals for the two gases, the ratio data stay remarkably constant, reflecting a constant oxygen concentration for well-ventilated sinuses in healthy subjects.

#### 3.2.2. Monitoring during One Day

**Figure 11:**
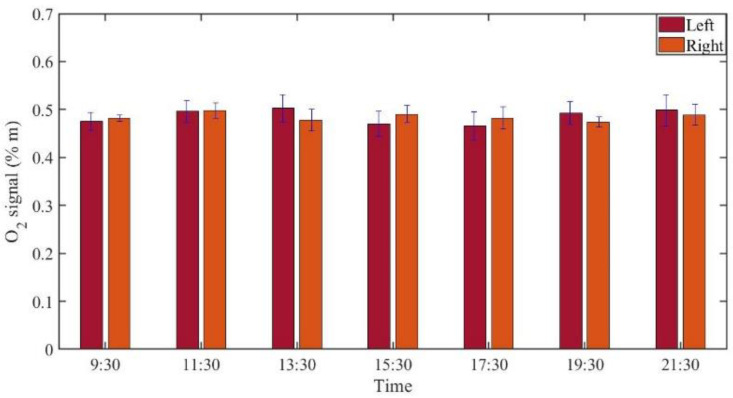
The changes of measured oxygen signal from a frontal sinus of the healthy volunteer 1 during one day.

**Figure 12:**
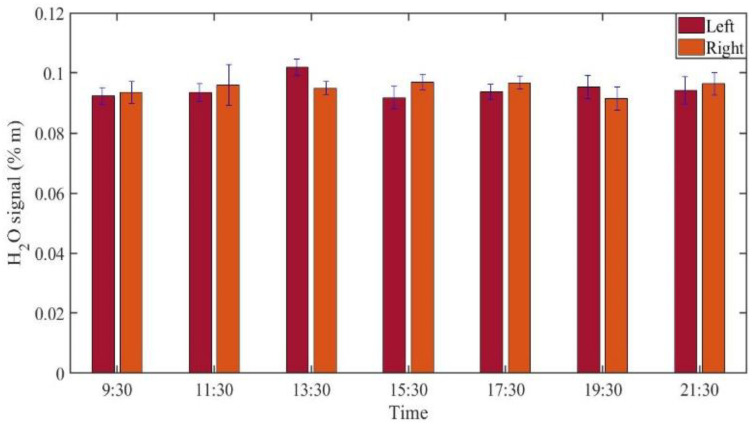
The changes of measured water vapor signal from a frontal sinus of the healthy volunteer 1 during one day.

**Figure 13:**
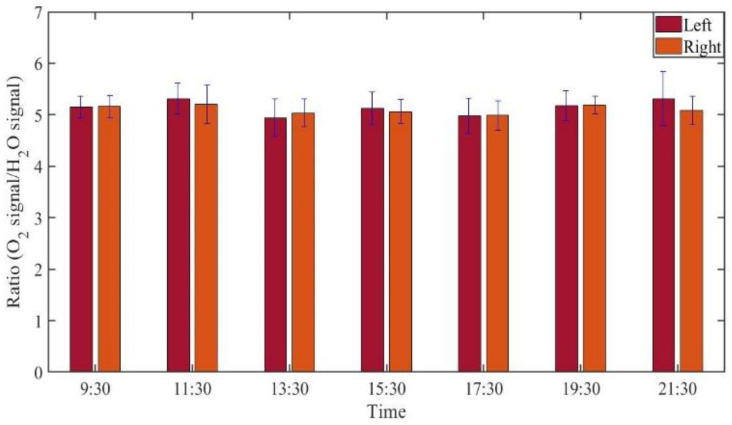
The ratio of measured oxygen signal to water vapor signal on volunteer 1 during one day.

GASMAS data might change somewhat from day to day, e.g., because of different filling with mucus and possible slow changes in health status. Such trends are observed in the data given in Figure 5, Figure 6, Figure 7, Figure 8, Figure 9 and Figure 10. In order to further assess the instrumental and methodological stability and reproducibility of the GASMAS technique applied in sinus monitoring, it was also considered valuable to follow the signals during a single day. For this study on volunteer 1, only one position was chosen, at the left and right sides of the face. Gas signals from the sinuses of this volunteer were measured every two hours throughout the day, starting at 9:30 a.m. and ending at 9:30 p.m. Data for oxygen, water vapor and evaluated ratio are given with one standard deviation in Figure 11, Figure 12 and Figure 13.

The figures show that the signals for both gases obtained from the volunteer are extremely stable, and correspondingly, the ratio is stable. The ratio data in Figure 7, Figure 10 and Figure 13 all give a value close to 5.2, with a very small error bar.

### 3.3. Oxygen Concentration Retrieval

The sinuses are not highly ventilated, so the temperature is expected to be close to 37 °C, since they are from all sides imbedded in blood-supplied tissue, and have limited ventilation flow. Further, the sinuses contain liquid water, leading to 100% relative humidity, again, since the ventilation is limited. As obtained from the Arden-Buck relation [31], the concentration of water vapor in the sinuses is then about 6.2%. According to Equation (3), by multiplying the signal ratio by the concentration of water vapor in the sinuses, the concentration of oxygen in the sinuses is obtained. According to Figure 7, Figure 10 and Figure 13, the ratio in the sinuses of all healthy volunteers is close to 5.2, a value which should be adjusted with the calibration factor 1.48, as obtained in Section 3.1. A true signal ratio of 3.5 is then found, and the resulting oxygen concentration 3.5∗6.2%≈22%, which is close to what is expected ((21+17)/2=19%) for sinuses with connection to the nose, where incoming air contains 21% and exhaled air contains 17% after the generation of 4% carbon dioxide in the lungs.

### 3.4. Gas Transport through Ostia

**Figure 14:**
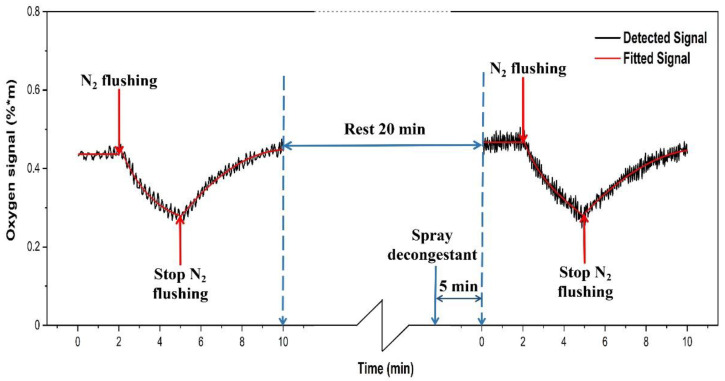
The measured O_2_ signal from the left frontal sinus of the healthy volunteer. The dynamic of gas exchange, in the absence and presence of decongestant, is shown.

Our gas transport study was performed using pure nitrogen flushed through the nostril of two volunteers, one without any symptoms, and one with a clear rhinitis condition. During the flow, the volunteers breathed through the mouth only, to ensure full oxygen supply to the lungs, while the nasal cavity had low oxygen. Sinus oxygen gas at 760 nm was monitored with a geometry corresponding to position II. The study for each volunteer was divided into two parts. Every gas exchange monitoring sequence lasted for 10 min. As shown in Figure 14, the first 60 recordings of the oxygen signal of the left frontal sinus of the healthy volunteer were taken during 2 min, to establish a background signal level. Then 90 recordings (3 min) were taken while a flow of pure nitrogen into the nostril was running. Finally, 150 recordings (5 min) were taken after stopping the flow of nitrogen.

After the end of the first part of the study, the healthy volunteer was asked to rest for 20 min. At the 15th minute, decongestant was sprayed into the left nostril of the volunteer. After 5 min of action, the second part of the study was conducted, and again lasted for 10 min. For 60 recordings (2 min), the background oxygen level was recorded for the same frontal sinus of the volunteer after spraying decongestant. Then 90 recordings (3 min) were taken during nitrogen flow. Finally, 150 recordings (5 min) of the oxygen signal were taken after the nitrogen flow had stopped. Figure 14 shows the results of the recordings on the healthy volunteer.

During the first part of the study, when flowing nitrogen through the healthy volunteer’s nostril, the oxygen signal decreases, indicating that the sinus ostium was open, and nitrogen could partly replace oxygen in the sinus. After stopping the nitrogen flow, the oxygen signal returns to its original value signal recording, since oxygen was again displacing nitrogen. Spraying the decongestant into the nostril of the healthy volunteer, it was noted that the baseline oxygen value was basically the same, and also the dynamic behavior on nitrogen flushing was the same. In both cases it was found that the fall-off and increase curves, when fitted with exponentials, remain indifferent with and without decongestant. The decay and increase curves have time constants of the order of 5 min.

**Figure 15:**
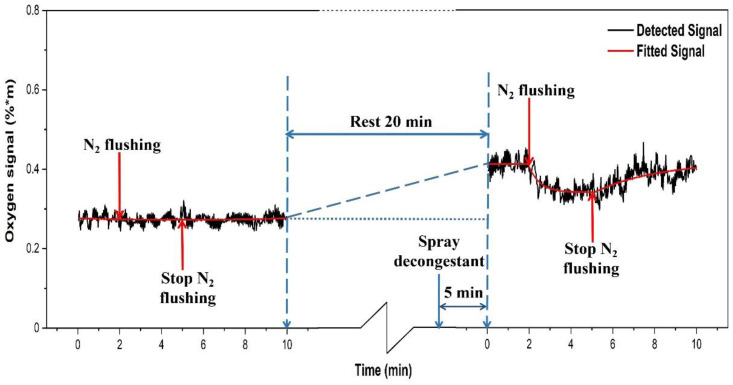
The measured O_2_ signal from the left frontal sinus of the rhinitis volunteer. The influence of spraying a decongestant is shown.

The corresponding data for the rhinitis volunteer, recorded in an identical measurement sequence, are shown in Figure 15.

When the rhinitis volunteer did not receive decongestant, the oxygen signal basically did not change when nitrogen was let in, indicating that the nasal passage was blocked. The baseline value is about 0.27, which is significantly lower than for the healthy volunteers, with an average value for position II of volunteer 1 of 0.53 (Figure 5) and 0.54 for volunteer 2 (Figure 8). When decongestant was sprayed in the volunteer with rhinitis, and after 5 min the oxygen signal was measured, it was found that it had increased by 51% to 0.41, indicating that the initially low oxygen content in the closed sinus now increased by fresh air influx through the opened ostium. When the nasal cavity was filled with nitrogen, the signal of oxygen decreased, verifying that the decongestant did open up the sinus nasal passage. However, now the maximum reduction was only 17% in contrast to 44% for the healthy volunteer. Our ventilation study did not require the parallel monitoring of water vapor, since the oxygen signals levels are internally compared in response to the nitrogen flushing.

## 4. Discussion and Conclusions

The water vapor signal is related to the size of the sinus, since the water vapor concentration is constant and well-defined. Clearly, it also strongly depends on the measurement geometry, which in our study was kept constant for each measurement site. The oxygen signal depends on the size as well as the oxygen concentration in the sinus. By dividing the oxygen and water vapor signals, the size of the volume as well as the influence of the geometry are eliminated, and the oxygen concentration can be determined.

Measurements on healthy volunteers, performed frequently during one day, and also every day during one week, demonstrated human frontal sinus signal consistent co-variation regarding water vapor and oxygen, using our non-invasive diode laser spectroscopy technique. Relative signal intensities for different monitoring sites largely persist as well as left-right asymmetries, showing that the technique provides highly stable reflections of sinus size/geometry. Small observed changes over time might reflect small difference in mucus volume. A laser at 760 nm was used for oxygen monitoring, and a laser at 818 nm (instead of at 937 nm) for water vapor monitoring was for the first time employed for the study of the frontal sinus gas signals. By moving the water vapor laser wavelength closer to that pertaining to oxygen, the differences in attenuation and scattering in the light propagation are strongly reduced, thus improving the accuracy in oxygen concentration retrieval using Equations (2) and (3). This is particularly valid for frontal sinus monitoring, where the back-scattering geometry is the only possible one, in contrast to the case for maxillary sinuses, where trans-illumination from the mouth to a detector on the skin can be used [37].

The ratio of the oxygen and water vapor signals was found to be particularly stable, reflecting a constant oxygen concentration in well-ventilated sinus cavities. For volunteer 1, the ratio (standard deviation) as evaluated from all the data in Figure 7 is 5.12 (0.03) for the left sinus, while we obtain 5.18 (0.11) for the right sinus. Corresponding values for volunteer 2 (Figure 10) are 5.23 (0.07) for the left sinus and 5.18 (0.08) for the right sinus. Likewise, the ratio was found to be 5.11 (0.10) for the one-day monitoring of a single sinus of volunteer 1. All these values closely coalesce. Using the global average 5.16 and applying the calibration factor 1.48 (Section 3.1), we, with an expected water vapor concentration of 6.2% (T = 37 °C, RH = 100%), arrive at a sinus average concentration of 21.6%. The value is close to the average concentration of inhaled and exhaled air, 21 and 17%, respectively. The deviation is easily explained by small inaccuracies in calibration and sinus temperature values (exponential dependence) and relative humidity (linear dependence). As an example, a temperature reduction of the sinus air of 1 °C would lead to an oxygen concentration reduction from 21.6% to 20.4%. We would arrive at the nominal concentration of 19 % if the effective sinus gas temperature was 35 °C.

Sinus cavities are seldom completely left-right symmetrical. This is also reflected in the measured data for the individual gases. Thus, from Figure 5 and Figure 6, the left/right ratio was found to be 0.91 (0.10) and 0.92 (0.09) for oxygen and water vapor, respectively, as average values for the three measurement locations on each side for volunteer 1. Corresponding left/right ratio values for volunteer 2, as obtained from Figure 8 and Figure 9, are 1.10 (0.05) and 1.09 (0.06), respectively. A high consistency between the two gases in the asymmetry assessment is thus found. As mentioned, the signal ratio, reflecting the oxygen concentration, should be and is found to be unaffected by asymmetry.

As a further observation from the data, the measurement positions I, II and III systematically lead to larger individual gas signals, reflecting the longer average passage through gas. The percentage increase averaged for both gases going from position I to II, and from I to III is 18 (2) and 34 (1)%, respectively, for volunteer 1, as evaluated from the individual day values. The corresponding values for volunteer 2 are 20 (5) and 31 (4)%, respectively. The different numbers for the two volunteers, together with the measured asymmetries, clearly reflect the topography of the individual cavities.

In conclusion, the internally very consistent results regarding various monitoring parameters, which were studied in the present work, give full confidence in the reliable operation of the GASMAS technique. The results also show that the weaker signals obtained when using the weaker 818 nm band yield sufficient signal quality to ensure an uncompromised evaluation of absolute oxygen concentrations.

Furthermore, our study showed that it is possible to objectively assess sinus ventilation through the ostium. The effect on the oxygen content of spraying decongestant of the frontal sinus of volunteers with and without rhinitis was studied. Nitrogen flushing before and after spraying decongestant was performed. The opening up of the passage with decongestant was objectively established for the first time, by observing the action of nitrogen flow into the sinus. This opens up for future investigations of the relative efficacies of different decongestant agents to be studied non-intrusively and objectively for the first time. Since the nasal passages of healthy volunteers are always open, we, as expected, did not see any significant influence of the decongestant spray.

Together with clinical partners, a study on patients is planned to establish the clinical value of the method. In particular, any relation between the type of bacteria and viruses found in patients suffering from sinus disorders to the GASMAS signal will be investigated, which may prove useful in sinusitis diagnostics and contribute in the abatement of the development of antibiotics resistance.

## Data Availability

Original data can be obtained on request from the authors.
